# A Global Analysis of the Effectiveness of Marine Protected Areas in Preventing Coral Loss

**DOI:** 10.1371/journal.pone.0009278

**Published:** 2010-02-17

**Authors:** Elizabeth R. Selig, John F. Bruno

**Affiliations:** 1 Curriculum in Ecology, University of North Carolina, Chapel Hill, North Carolina, United States of America; 2 Department of Marine Sciences, University of North Carolina, Chapel Hill, North Carolina, United States of America; Institut Pluridisciplinaire Hubert Curien, France

## Abstract

**Background:**

A variety of human activities have led to the recent global decline of reef-building corals [1], [2]. The ecological, social, and economic value of coral reefs has made them an international conservation priority [2], [3]. The success of Marine Protected Areas (MPAs) in restoring fish populations [4] has led to optimism that they could also benefit corals by indirectly reducing threats like overfishing, which cause coral degradation and mortality [2], [5]. However, the general efficacy of MPAs in increasing coral reef resilience has never been tested.

**Methodology/Principal Findings:**

We compiled a global database of 8534 live coral cover surveys from 1969–2006 to compare annual changes in coral cover inside 310 MPAs to unprotected areas. We found that on average, coral cover within MPAs remained constant, while coral cover on unprotected reefs declined. Although the short-term differences between unprotected and protected reefs are modest, they could be significant over the long-term if the effects are temporally consistent. Our results also suggest that older MPAs were generally more effective in preventing coral loss. Initially, coral cover continued to decrease after MPA establishment. Several years later, however, rates of coral cover decline slowed and then stabilized so that further losses stopped.

**Conclusions/Significance:**

These findings suggest that MPAs can be a useful tool not only for fisheries management, but also for maintaining coral cover. Furthermore, the benefits of MPAs appear to increase with the number of years since MPA establishment. Given the time needed to maximize MPA benefits, there should be increased emphasis on implementing new MPAs and strengthening the enforcement of existing MPAs.

## Introduction

A variety of human activities have caused the recent global decline of reef-building corals [1], [2], [6]. Coral loss has cascading effects throughout reef ecosystems leading to subsequent changes in the population dynamics of reef inhabitants [7], [8]. In spite of their socio-economic and ecological importance, [2], [3], we have few proven solutions and tools to enable local and regional managers to mitigate coral loss.

By limiting or preventing fishing and other extractive activities, Marine Protected Areas (MPAs) have been relatively successful in restoring populations of overharvested fish and invertebrates [4]. The success of tropical MPAs in protecting fish [9] has led to optimism that they may also have positive, indirect effects on corals [2], [5]. MPAs could benefit corals indirectly by preventing overfishing and restoring coral reef food webs [3], [6], [10]. More intact food webs could prevent outbreaks of coral predators [11] and, in some cases, may limit the coverage of macroalgae by restoring grazer populations, which could in turn facilitate coral recruitment [12], [13]. More directly, MPAs could prevent destructive fishing practices, anchor damage, and terrestrial run-off if they include a terrestrial component that reduces sedimentation and nutrient pollution.

However, protection within MPAs may not necessarily result in positive effects on coral cover. Coral loss that is driven by regional or global stressors like climate change and coral disease outbreaks seems unlikely to be mitigated by MPAs or other local management actions [14], [15]. Indeed, several studies of individual reefs or small groups of reefs have found that MPAs do not prevent coral loss and other forms of reef degradation [7], [15], [16], [17].

In spite of the importance of reducing coral losses, no global analyses have explored the potential role of MPAs in reducing coral decline. Coral cover, or the percentage of hard substrate covered by living coral tissue, is a key measure of coral ecosystem health. We compiled a global coral cover database to determine whether changes in benthic coverage by living scleractinian (stony) corals differed within MPAs compared to unprotected reefs. We also examined the potential influence of location (ocean basin) and years since MPA implementation on the mitigation of coral loss by MPAs.

## Methods

**Figure 1:**
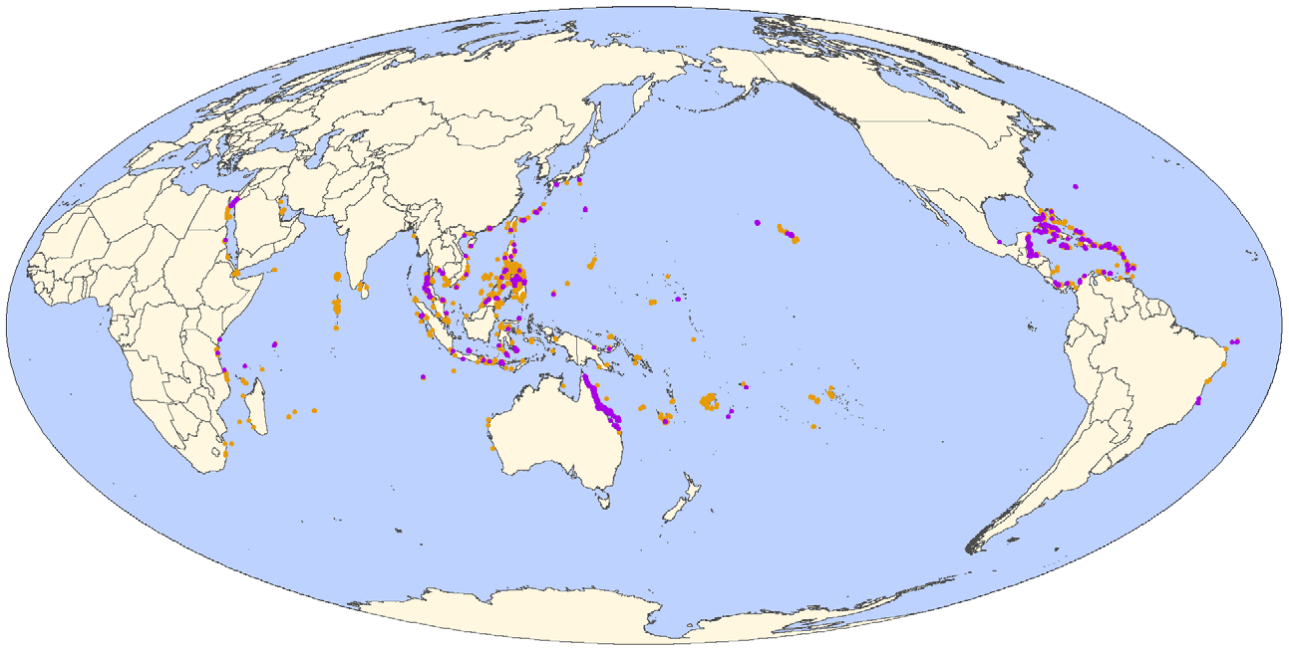
Location of unprotected (orange) and protected (purple) reef coral cover survey sites.

We compiled a comprehensive global database to compare long-term changes (1969 to 2006) in coral cover from 5170 independent surveys inside 310 MPAs around the world to 3364 surveys of unprotected reefs (Fig. 1). Surveys were from 4456 reefs across 83 different countries, although a few well-surveyed countries were more represented in the dataset. For example, of the 8534 surveys that were conducted globally, 2025 surveys were from the Great Barrier Reef. We had 993 surveys that were repeated at least twice with 306 in the Caribbean and 687 in the Indo-Pacific. When we compiled our database, reef surveys were included regardless of the purpose of the study. The MPAs in our analysis covered a large range of ages, sizes and degrees of enforcement (Text S1; Fig. S1). The surveys in the database were conducted across more than 40 years in the Caribbean and more than 30 years in the Indo-Pacific. This long temporal range allowed us to compare coral cover with the number of years of protection at the time of the survey.

We constructed different multi-level models to compare changes in coral cover over time between MPA and non-MPA reefs and to determine how the number of years of protection in MPAs affected temporal changes in coral cover. Multi-level models use parameters that can vary at more than one level. We used this approach to incorporate the spatial and temporal structure (i.e. spatial clustering and repeated sampling on some reefs) in the coral cover data. We then determined the necessary random effects and additional predictors to incorporate into the model using Akaike Information Criterion. Because change in coral cover is often dependent on initial coral cover [18], we estimated coral cover change using estimated cover in the previous year for each year in all of our models. Therefore, the difference in change in coral cover in protected versus unprotected areas can vary by year according to our data (Text S1).

For the ‘MPA versus non-MPA model’, we grouped protected reefs within each MPA with all unprotected reefs within 200 km (Fig. S2). This approach allowed us to compare the trends within each MPA to the population of ‘control’ reefs within the 200 km buffer rather than selecting a single unprotected reef, which could introduce site selection biases and a variety of other problems [19], [20]. We grouped MPA surveys with non-MPA surveys so that we maximized our sample size (the number of possible groupings of MPA and non-MPA surveys) without greatly increasing the variability within the grouping. As distance from the MPA reefs increases, reefs are likely to be experiencing different environments and could also be compositionally and structurally distinct. Including more distant reefs could therefore increase heterogeneity and variability with the spatial group enough to make it difficult to detect an effect of protection. We used loglikelihood analysis to determine that an optimal distance for grouping MPAs with non-MPAs was 200 km (Fig. S2; Text S1).

For the ‘years of protection model’, which only included surveys on reefs within MPAs, we built two sub-models: one for the protected Caribbean reefs and the other for all the protected reefs in the Indian and Pacific Oceans (hereafter Indo-Pacific). We used these models to assess whether the number of years of protection affected changes in coral cover within MPAs. When we explored different model forms using generalized additive mixed models for the two ocean basins, we found that a linear model was sufficient for the Caribbean (Fig. S5A), but a non-linear changepoint or breakpoint model [21] was needed for the Indo-Pacific (Fig. S5B).

**Figure 2:**
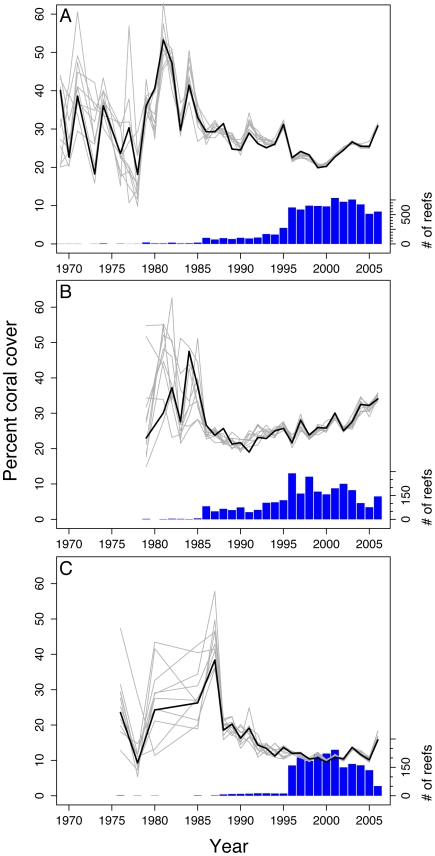
Comparisons of the average coral cover per year as predicted by the models. Simulated data sets (light grey lines) with the observed mean coral cover per year (thick black line) for (**A**) MPA versus control model, (**B**) MPA-only Caribbean years of protection model, and (**C**) MPA-only Indo-Pacific years of protection model. The histograms at the bottom of the figures display the relative sample sizes at each year for the actual data. In all models, the earlier years had less data and therefore exhibit more variation in behavior. In the ‘year of protection’ models there was not sufficient data to accurately estimate percent coral cover so the simulation results begin in later years. Note that the right y-axes are different in each of the plots due to the varying number of observations in each model.

The final AIC-recommended models for both the MPA versus non-MPA model and the years of protection models for the Caribbean and Indo-Pacific were refit as Bayesian models to obtain more realistic estimates of parameter precision [22; Figs. S3, S4, Text S1]. We calculated rates of change in coral cover for individual reefs and MPAs as well as population-averages across all reefs and MPAs using the final multilevel statistical models (Figs. 2, S4) and calculated *R* ^2^ for both (Table S1–Table S2). These calculations allowed us to examine trends across reefs and to compare the modeled patterns to observed data (Fig. 2).

Most local studies of MPA efficacy compare population or community parameters within the protected area to a nearby, unprotected control site. Although these single-site studies can be powerful tests of the efficacy of a single MPA, there can be biases caused by MPA site selection or problems identifying suitable independent control sites that make interpreting and generalizing the results of such studies problematic [19], [20]. The multi-level modeling approach allowed us to investigate both individual and population-average reef and MPA trends, while taking into account the temporal and spatial structure in the data (Fig. 2). With our analysis, we fit the model to all the available data even for those reefs that were surveyed once or only a few times [23]. Calculating these population-averages is particularly important for determining regional scale patterns because long-term monitoring data do not exist globally for many reefs. Locations with more long-term data are weighted more heavily, but even reefs with one survey were also able to be included in the model. To test whether the MPAs that we included in our analyses were not preferentially located on reefs that were naturally more resilient, i.e. a site-selection bias, we compared coral cover on MPA and non-MPA reefs within the first five years of MPA establishment.

## Results and Discussion

**Figure 3:**
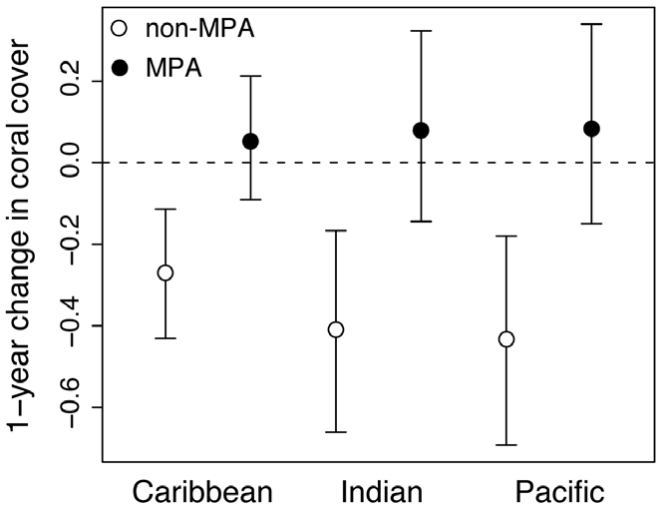
The change in percent coral cover from 2004 to 2005 inside and outside of MPAs. The 95% credibility intervals (error bars) are also shown. Reefs protected in MPAs had slightly positive changes in percent coral cover, although not significantly different from zero (dashed line). Percent coral cover was obtained by back-transforming the predicted logit from the model.

We found that MPAs can be effective in preventing coral losses. There was no change in coral cover over time across all reefs within MPAs over 38 years. In contrast, coral cover on unprotected reefs continued to decline throughout this period. Our analyses also enabled us not only to examine these overall long-term patterns (Fig. 2), but also the difference in coral cover change in protected versus unprotected reefs for individual years based on the modeled percentage coral cover in the previous year (Fig. 3). For example, from 2004 to 2005, the most recent, complete year in our database, coral cover within MPAs increased by 0.05% in the Caribbean and 0.08% in the Pacific and Indian Oceans (Fig. 3). In contrast, average declines on unprotected reefs from 2004–2005 ranged from 0.27% in the Caribbean to 0.41% and 0.43% in the Indian Pacific Oceans, respectively (Fig. 3). Although the year-to-year changes in coral cover may seem trivial over the short-term, the cumulative effects could be substantial over several decades.

**Figure 4:**
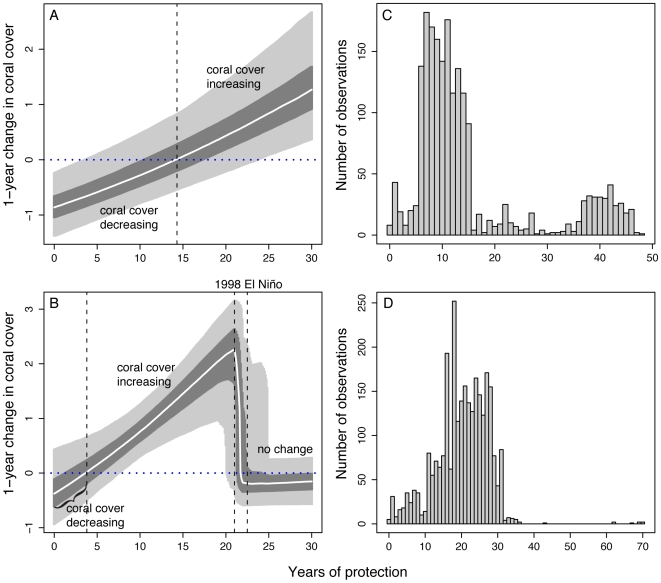
The effect of the number of years of protection on the 1-year change in coral cover and number of observation. Coral cover change rates are shown in the (**A**) Caribbean and (**B**) Indo-Pacific with the 95% credibility intervals (light grey bands) and the 50% credibility intervals (dark grey bands) as well as the median (white line) of the posterior distributions of the year of protection models using all years of data and 2005 coral cover change rates. The number of years of protection by observations (surveys) in the (**C**) Caribbean and (**D**) Indo-Pacific show that most surveys have been performed when MPAs have been established for 15 years or less.

The effectiveness of MPAs in preventing coral loss was strongly dependent on the duration of protection. This finding is consistent with previous work on commercial fish stocks in Europe and southern Australian reef communities that found that the positive effects of MPAs increased with the number of years of protection [24], [25]. We calculated the relationship between the 1-year change in coral cover and the number of years of protection (Fig. 4). In the Caribbean, coral cover continued to decline for approximately 14 years after protection began (Fig. 4A), possibly due to the time required for fish stocks to rebound from previous exploitation [26]. Coral cover change rates then stopped declining and began to increase with the number of years since MPA implementation (Fig. 4A). The coral cover change rates also began to exhibit some leveling-off as the number of years of protected became greater, a finding that is consistent with several studies of protection on reef fish [26], [27], [28] and at least one field study on coral cover [29], which found that population recovery reached a saturation point. Because many reef-building corals are slow-growing, recovery rates and coral cover change will be influenced by the life history and growth rates of locally dominant species. A rebound in coral cover may also reflect a shift to faster-growing or more stress-tolerant species or genotypes and a subsequent change in species composition [30].

The effects of MPA duration were somewhat different in the Indo-Pacific. Coral cover inside Indo-Pacific MPAs continued to decline for the first 5 years following MPA implementation. Coral cover then began to increase to relatively high rates of approximately 2% annually until around 22 years of protection (20.0–24.9 years 95% credibility interval; Fig. 4B). This strong, positive effect of MPAs on coral recovery ended after two decades of protection (Fig. 4B). This decline or “reset” coincided with a cohort of reefs that had been protected for 20–25 years when the strong El Niño of 1998 occurred (Fig. 4B, 4D). The 1998 El Niño caused high coral mortality across the Indo-Pacific, even on reefs within MPAs [7], [15], [31]. This result supports previous studies that indicate that MPAs may not always protect corals from broad-scale natural and anthropogenic disturbances such as ocean warming [15], large storms and outbreaks of diseases [14], [32]. In spite of the decrease in MPA benefits following these severe disturbance events, our finding suggest that older MPAs do increase overall coral resilience because the rate of change in coral cover returns to change rates not significantly different from zero (Fig. 4B).

The creation of MPAs is usually based on complex negotiations among a variety of stakeholders including scientists, managers, politicians, conservation groups and fishers that have not previously used conservation criteria as the primary data for selecting MPA locations [33], [34], [35], [36]. Unfortunately, few MPAs have documents that report the guidelines that were used in the delineation of reserve boundaries, but it seems reasonable to assume that to some degree, reefs are often selected for protection based on some attribute that elevated their conservation value relative to other candidate reefs, i.e., high coral cover or proximity to a tourist area. Bias in MPA site selection could influence the detection of a positive MPA effect because MPAs may have initially been located where reefs were “healthier” or less frequently disturbed by natural disturbances, e.g., by storms. On the other hand, the political complexities of creating MPAs can also select for places that are less optimal for conservation because they are placed in areas that have less conflict with local fishers or other economic interests [25], [34]. Similarly, “unhealthier” reefs may have been selected for protection because of attempts to restore previously healthy ecosystems [37] or fisheries [38].

More recent strategies for selecting sites for MPAs have focused on maximizing conservation goals while creating a participatory process that addresses the concerns of a variety of stakeholders [35]. However, to our knowledge, these processes have only been implemented within the last 5 years for most of our study areas [35], [39]. Even for The Great Barrier Reef Marine Park, which changed its zoning in 2004 to increase connectivity and the percent of reefs in no-take areas [39], nearly all of our survey data pre-dated the new zoning. Complementing these approaches are also current efforts to identify and protect reefs that exhibit some degree of natural resistance or resilience to disturbances such as climate change [40], [41]. However, there is not yet evidence that sites based on these criteria are outperforming other areas, although there may not have been sufficient time to detect a positive effect.

We included data in our analyses without regard to the purpose of the study or the location of the MPA. With the large sample size of our analyses, biases in site selection would have to have been occurring not only across years, but also across countries with different management goals and socioeconomic structures. Ideally, we could test for biases by looking at differences in coral cover before and after the establishment of MPAs [20]. However, there are very few cases where this type of monitoring has been done. As an alternative approach, we compared the percent coral cover within the first five years of MPA establishment and found no significant difference between coral cover in MPAs (mean = 32.4) versus non-MPA (mean = 30.2) (Welch Two Sample t-test; t = −0.5007, df = 45.47, p-value = 0.69). This finding suggests that there should be no bias in our results because of differences in the initial percent coral cover values inside and outside of MPAs.

Several recent studies of individual reefs or groups of reefs at broader scales have failed to find a positive effect of MPA on coral cover [7], [15], [16], [17]. Indeed, previous research indicates that there can be substantial reef-to-reef heterogeneity at local scales [42], [43], which may make it difficult to detect an effect of protection. Relatively small sample sizes in some of these studies may have meant that there was too little power to detect positive effects on coral cover. Comparatively small annual effects and the short duration of most single MPA versus non-MPA comparisons may also have complicated efforts to find an MPA effect. Although we also found relatively subtle differences between the annual coral cover rates between MPA and non-MPAs area, the cumulative benefits over time could be quite substantial. For example, over 30 years, if coral reefs in the Indo-Pacific continued to decline at approximately 0.4% per year as they did in 2005 (Fig. 3), hypothetically an additional 12% coral cover would be lost whereas coral cover on protected reefs could remain relatively unchanged.

Our results may even be a conservative estimate of MPA benefits because many tropical MPAs have poor enforcement of their regulations [44] and most MPAs have only recently been established [45]. Some of the reefs we categorized as being in MPAs are probably essentially unprotected. Levels of enforcement are rarely quantified or reported, so we could not exclude poorly managed MPAs from our analyses or include the degree of enforcement as a covariate in our statistical models. Almost 60% of the surveys in our analysis were from MPAs that were less than 15 years old (Figs. 4C and 4D). Since benefits may increase with MPA age, the general benefit of MPAs could be greater than our estimates. In addition, only 13.4% of reefs are currently protected in non-extractive or multi-use MPAs and only 1.4% are in no-take reserves [45]. Protecting a greater percentage of reefs could lead not only to increased coral cover, but also to positive, synergistic effects of having more connected populations protected. Regardless, assessing the capacity of the current MPA network to improve coral reef condition is important for galvanizing future efforts to tighten enforcement and expand the overall area of protected reefs.

MPAs can play a critical role in the protection of coral reef ecosystems, particularly fisheries [4], [10]. Our results suggest that MPAs are also generally effective in reducing or preventing coral loss. Nonetheless, we were not able to assess their effects on other metrics of reef health including changes in other key taxonomic species [46], coral composition, richness, reef heterogeneity and other factors that could also indicate that there has been a decline in reef health [47], [48], [49]. MPA benefits may appear modest in the short term, but over several decades could lead to large and highly ecologically significant increases in coral cover as the cumulative importance of small annual effects becomes more important and the number of years of MPA protection increases. However, it remains to be seen whether the observed benefits of MPAs are sufficient to offset coral losses from major disease outbreaks and bleaching events, both of which are predicted to increase in frequency with climate change [18], [50]. Given the time lag for maximizing MPA effectiveness, implementing new MPAs and increasing enforcement should help maximize the ability of MPAs to prevent future coral loss.

## Supporting Information

Text S1 Supporting methods
(0.11 MB DOC)

Figure S1 The number of reefs by the year of MPA establishment for the (A) Caribbean and (B) Indo-Pacific.
(0.58 MB TIF)

Figure S2 The relationship between the MPA effect on slope (change in coral cover) and the distance of non-MPAs surveys from MPAs. The loglikelihood (solid black line) is maximized at 200 km, where approximately 60% of the non-MPA data has been paired in a structural unit with MPA data (dashed green line). MPA effect on slope and confidence intervals (grey dashed line) do not vary significantly with distance.
(0.54 MB TIF)

Figure S3 AIC values for all models examined. The best model is the one with the smallest AIC value. In this case, the best model is one in which MPA modifies the slope and intercept and ocean modifies the intercept only. Models with AICs that exceed 18650 are designated with arrows.
(0.39 MB TIF)

Figure S4 Coefficient estimates for the MPA versus non-MPA model. The 95% credibility intervals (thin light grey line) and the 50% credibility intervals (thick dark grey line) as well as point estimates (median) of the posterior distributions for all parameters in the MPA versus non-MPA model using a Bayesian approach to fit the model. There is a 95% probability that the true value lies within the 95% credibility interval. The MPA x 10-Year Trend term should be contrasted with the 10-Year Trend term, which is the trend for non-MPAs. The MPA x 10-Year Trend term is an effect and gets added to the 10-Year Trend term when MPA = 1 to obtain the trend for MPAs.
(0.41 MB TIF)

Figure S5 Generalized additive mixed models (non-parametric estimation) for the (A) Caribbean and (B) Indo-Pacific. There is no evidence of a changepoint in the Caribbean, but there is in the Indo-Pacific. The 95% confidence intervals are shown with dashed lines. The models have been smoothed with a 5-year running mean.
(0.55 MB TIF)

Table S1 *R^2^* for MPA versus non-MPA model. NA denotes the lack of a predictor for the calculation of *R^2^*.
(0.02 MB DOC)

Table S2 *R^2^* for MPA-only models in the Caribbean and Indo-Pacific. *R^2^* can only be calculated at level 1 for these models.
(0.02 MB DOC)
