# Supplementary material for: A Global Analysis of the Effectiveness of Marine Protected Areas in Preventing Coral Loss
Source: PLoS One. 2010 Feb 17;5(2):e9278. doi: 10.1371/journal.pone.0009278 (PMC2822846; doi:10.1371/journal.pone.0009278)
Supplement: Text S1 — Supporting methods (0.11 MB DOC) [file pone.0009278.s001.doc]

SUPPORTING INFORMATION

A global analysis of the effectiveness of marine protected areas

in preventing coral loss

Elizabeth R. Selig and John F. Bruno

**Text S1**

*Coral and MPA databases*

We developed a global database of 8534 surveys that quantified the percentage of the shallow water hard substratum covered by living hard (scleractinian) coral tissue on 4456 reefs from 1969 to 2006 [1]. Surveys on 993 of 4456 reefs were repeated at least twice. Of the repeatedly monitored reefs, 386 had five or more observations. Data were collected from a variety of sources including peer-reviewed literature, published government and non-governmental organization reports, and existing databases (i.e., Reef Check, Reefs at Risk in Southeast Asia, ReefBase). We used the ISI Web of Science and Google Scholar to find appropriate peer-reviewed and grey literature from management agencies and environmental organizations, targeting our search with the terms "coral" and "cover" and "reef" and "health". We also examined all available issues of journals that were likely to contain coral reef studies including *Atoll Research Bulletin*, *Coral Reefs*, *Marine Pollution Bulletin*, and the *Proceedings of the International Coral Reef Symposium*. Only surveys above 15 m depth were included to avoid depth biases [for more detail on surveys in the Indo-Pacific regions, see 1]. We included surveys without any biases as to the purpose of the study and most surveys were not carried out to measure. Therefore we believe we were able to make broad comparisons and generalizations about MPA effectiveness with a relative lack of biases.

The World Database on Protected Areas (WDPA) maintains a freely available spatial dataset on protected areas worldwide (<http://sea.unep-wcmc.org/wdbpa>) including data on the date of establishment (Figs. 4C, 4D, and S1). For the Caribbean, some protected areas were updated with data from The Nature Conservancy. In addition, boundaries for the United States' national marine sanctuaries, monuments, and parks were obtained from the National Oceanographic and Atmospheric Administration, the US Department of the Interior, or the United States Geological Survey where available. We also obtained exact boundaries from the Great Barrier Reef Marine Park Authority for the Great Barrier Reef Marine Park for the pre-2005 zoning, which was the management plan under which all of our data was collected except for one year. Even MPAs which did not have polygon boundaries had information about their total area. For the final analysis, 27 of the 310 total MPAs analyzed lacked exact boundary information. In these cases, we followed the protocol of similar work [2] and delineated a circular buffer around the point to create a boundary of the stated area of the MPA.

***Multilevel model development***

We used a multilevel model to examine the relationship between live coral cover and protection over time. Because some surveys were repeated over time, the data could not be considered independent. In addition, reefs were spatially clustered and this autocorrelation had to be accounted for in the statistical model. By using a multilevel model, we were able to include such spatial and temporal structure explicitly [3]. This approach focused on patterns at the scale of individual reefs while still providing population averages for reefs at regional scales [3]. We used the nlme library [4] in R 2.5.1 [5] and WinBUGS 1.4.3 [6] to analyze our models.

Percent coral cover is bounded data, which could be described by various probability models including the beta, logistic-normal, and arcsine-normal distributions. We applied a logit transformation to percent coral cover to obtain a logistic-normal distribution, which we then treated as normally distributed [7]. We also centered our time regressor in the models we examined. Centering not only reduced correlation between the random effects and facilitated model convergence, but also made the intercept interpretable as the coral cover in the year selected. We found that centering the models on the year 1996 yielded the least degree of correlation between the random effects [8,9].

We began model development by constructing a generic multilevel model to account for the spatial and temporal correlation inherent in our data. The assignment of random effects in a multilevel model accounts for unexplained spatial and temporal heterogeneity. In our model, there were three possible levels, each of which separate observation errors from temporal and spatial effects. At level 1, individual observations on a reef were assigned a unique random effect. Surveys on the same reef over time were assigned a unique random effect at level 2, accounting for temporal correlation. Level 3 contained the spatial unit where protected and unprotected reefs were paired, accounting for spatial correlation.

The spatial units for level 3 were constructed based on MPA boundaries. All reefs within an MPA with the same management criteria were grouped together. Then we paired unprotected reefs with protected reefs based on their distance to the nearest MPA boundary that included a survey to create reasonably homogenous spatial grouping units. This definition exercise is analogous to trying to identify the appropriate blocking unit to which "treatments" (MPA versus non-MPA) are applied in an ANOVA context. These spatial groupings were used to assign random effects in level 3. We selected the pairing distance using maximum likelihood estimation to compare how a range of 13 distances between 0 and 4000 km influenced the MPA effect on the slope (coral cover change). At a distance of zero, each spatial unit or block had a single treatment, MPA. When the distance was greater than zero, non-MPA surveys were added to the spatial unit or block. Non-MPA surveys that were not close enough to be paired with an MPA area were assigned to their own spatial unit. We would expect that by increasing block size a treatment effect would become easier to detect in part due to the greater power achieved by a larger sample size. On the other hand, increasing the size of the spatial unit could increase variability within the block by adding dissimilar areas. These features suggest that there should be some optimal choice for distance pairing. The shape of our loglikelihood curve was concave down (Fig. S2) with a well-defined maximum, indicating that there was indeed an optimal distance value; the loglikelihood was maximized at a pairing distance of 200 km (Fig. S2). Reefs within 200 km of an MPA were paired with the nearest MPA that contained a coral cover survey; approximately 60% of reefs were paired. Any remaining unprotected or protected reefs were left unpaired but grouped into spatial units. For the MPA-only analyses (see below), the spatial groupings were the MPAs.

We began with an ordinary regression model with logit-transformed coral cover (hereafter, logit coral cover) as the response and centered year as the only predictor. We then added temporal structure and spatial structure and used information-theoretic measures (AIC) to conclude that we needed to account for both [10,11]. The basic trend model fit to each reef is shown in Eqn. (1). In this model, *Y* is logit coral cover, *T* is year, $\text{T}_{\text{c}}$ is the centering constant, and $\varepsilon$ is the random error. The subscript *i* denotes the spatial grouping unit, *j* denotes the reef within that spatial unit, and *k* denotes the individual survey measurement for that reef.

Level 1: $Y_{ijk}= \beta_{0ij}+ \beta_{1ij}\left(T_{\text{ijk}}- \text{T}_{\text{c}}\right)+ \varepsilon_{ijk}, \varepsilon_{ijk}\sim\text{N}\left(0,\sigma^{2}\right)$ (1)

The presence of the subscript *j* in the parameters $\beta_{0ij}$ (intercept) and$\beta_{1ij}$ (slope) indicates that these parameters were allowed to vary from reef to reef. The manner in which they vary is described by the level 2 equation, Eqn. (2).

Level 2: $\begin{matrix} \beta_{0ij}= \beta_{0i}+ \beta_{2i}X_{ij}+ u_{0ij} \\ \beta_{1ij}= \beta_{1i}+ \beta_{3i}X_{ij}+ u_{1ij} \end{matrix}$ , $\left[\begin{matrix} u_{0ij} \\ u_{1ij} \end{matrix}\right]\sim\text{N}\left(\left[\begin{matrix} 0 \\ 0 \end{matrix}\right],\left[\begin{matrix} \tau_{0}^{2} & \tau_{01} \\ \tau_{01} & \tau_{1}^{2} \end{matrix}\right]\right)$ (2)

Here, *X* is a reef-level predictor that varies among reefs, but is constant for observations taken on the same reef; $u_{0ij}$and $u_{1ij}$are the random effects for reef *j* in spatial grouping unit *i* and are assumed to have a joint normal distribution as shown. Protection status (protected versus unprotected) is an example of variable *X*. Random effects from different reefs were assumed to be independent and also independent of the level 1 error terms. The subscript *i* on the slope and intercept terms in Eqn. (2) indicates that reefs coming from the same spatial grouping unit may also have characteristics in common.

Level 3: $\begin{matrix} \beta_{0i}= \beta_{0}+ \beta_{4}Z_{i}+ v_{0i} \\ \beta_{1i}= \beta_{1}+ \beta_{5}Z_{i}+ v_{1i} \end{matrix}$, $\left[\begin{matrix} v_{0i} \\ v_{1i} \end{matrix}\right]\sim\text{N}\left(\left[\begin{matrix} 0 \\ 0 \end{matrix}\right],\left[\begin{matrix} \omega_{0}^{2} & \omega_{01} \\ \omega_{01} & \omega_{1}^{2} \end{matrix}\right]\right)$ (3)

In Eqn. (3), *Z* is a level 3 predictor and the terms $v_{0i}$and $v_{1i}$are the error terms. Examples of the variable *Z* include ocean basin. For all models, we determined through AIC that we should include random intercepts and random slopes at levels 2 and 3, which allowed coral cover over time and coral cover in a given year to vary among spatial grouping units as well as among reefs within those units.

***MPA versus non-MPA model***

We began with the basic form of the multilevel model described in Eqns. (1), (2), and (3) and added necessary predictors and spatial structure. As a result of the pairing, the original status of the reef, protected or not, varied with the spatial grouping unit. We treated protection status as a level 2 variable, which could be used to model the level 1 intercept, slope, or both, playing the role of variable $X_{ij}$ in Eqn. (2). Then we fit all possible models that included MPA as a level 2 predictor. Once we selected the appropriate MPA versus non-MPA model using AIC, we explored whether adding a variable that distinguished between ocean basins was needed as a level 3 predictor (Fig. S3). With a model that incorporated protection status as a level 2 predictor, we could determine whether the trend in coral cover varied between protected and unprotected reefs over time.

The best model for analyzing the differences in coral cover trends over time between protected and unprotected reefs included random effects for both the change in coral cover (slope) and intercept at levels 2 and 3. *Protection status* was a significant predictor of both the slope and intercept (*P* < 0.001). We also found that we needed an additional variable (level 3 predictor), which accounted for the ocean basin of the survey—Pacific Ocean, Caribbean Sea or Indian Ocean. The ocean variable was only needed to modify the intercept (Fig. S3 lists the models we fit and compares them using AIC). The AIC best model is shown in Eqn. (5):

$\begin{matrix} \mathrm{logit} \left(p_{ijk}\right)= \beta_{0}+ v_{0i}+\beta_{2}Protection Status_{ij}+\beta_{4}Indian_{i}+ \beta_{5}Pacific_{i}+u_{0ij} \\ + \left(\beta_{1}+v_{1i}+\beta_{3}Protection Status_{ij}+u_{1ij}\right)\left(T_{ijk}-\text{T}_{\text{c}}\right)+\varepsilon_{ijk} \end{matrix}$ (5)

Protection in MPAs resulted in a positive effect on the population-average trend (Fig. 3, S4). Change in coral cover was slightly positive but not significantly different from zero in MPAs and negative outside of MPAs (Figs.3, S4). The model explained 46% and 45% of the variation in intercept and slope, respectively, at level 2 and 39% of the variation in intercept at level 3 (Table S1).

***MPA-only model***

The MPA-only model used reefs inside MPAs to determine how the number of years of protection affected coral cover and its trend over time. The number of years of protection was a dynamic variable defined as the number of years the reef had been protected at the time of the coral cover survey. As with the MPA versus non-MPA model, we began with the basic model described by Eqns. (1), (2), and (3) that included random intercepts and random slopes at levels 2 and 3. We used AIC to compare whether adding a variable for the number of years of protection improved the model [10] either by modifying the slope or intercept. Because the number of years of protection at the time of the coral cover survey varied by the individual measurement on a reef, it was modeled as a level 1 variable. We then determined whether adding an additional variable for ocean basin was appropriate.

The number of years of protection variable significantly improved the model when added as a level 1 predictor. To better capture any ocean differences, we developed two models: one for the Caribbean and one for the Indian and Pacific Oceans (hereafter Indo-Pacific). When we explored different model forms using generalized additive mixed models, we found that a linear model was sufficient for the Caribbean (Fig. S5A), but a changepoint or breakpoint model [12] was needed for the Indo-Pacific (Fig. S5B). For the Caribbean, we fit the following linear model (Eqn. 6):

$\begin{matrix} \text{logit}(p_{ijk})=\beta_{0}+\beta_{2}Y_{ijk}+v_{0i}+u_{0ij} \\ +\left(\beta_{1}+\beta_{3}Y_{ijk}+v_{1i}+u_{1ij}\right)\left(T_{ijk}-\text{T}_{\text{C}}\right)+\varepsilon_{ijk} \end{matrix}$ (6)

In the Indo-Pacific changepoint model, the years of protection effect on trend was different for early and late years. Our final model for the Indo-Pacific (Eqn. 7) described years of protection having a different effect on trend depending on whether the number of years of protection occurs before or after the changepoint:

$\begin{matrix} \text{logit}(p_{ijk})=\beta_{0}+\beta_{2}Y_{ijk}+v_{0i}+u_{0ij} \\ +\left(\beta_{1}+\beta_{3}Y_{ijk}+\beta_{4}\left(Y_{ijk}-\text{Y}_{\text{CP}}\right)^{+}+v_{1i}+u_{1ij}\right)\left(T_{ijk}-\text{T}_{\text{C}}\right)+\varepsilon_{ijk} \end{matrix}$ (7)

Here *T* is the year, *Y* is the number of years the reef had been protected at the time of the coral cover survey, $\text{T}_{\text{C}}$ is the centering constant for years, $\text{Y}_{\text{CP}}$ is the breakpoint for years protected, and $\left(Y-\text{Y}_{\text{CP}}\right)^{+}$ is the indicator function defined as follows.

$\left(Y-\text{Y}_{\text{CP}}\right)^{+}=\left\{\begin{matrix} 0, & \text{if} Y-\text{Y}_{\text{CP}}\leq0 \\ Y-\text{Y}_{\text{CP}}, & \text{if} Y-\text{Y}_{\text{CP}}>0 \end{matrix}\right.$ (8)

We found that the Caribbean and Indo-Pacific models explained 91% and 81% of the variation at level 1, respectively (Table S2). To obtain parameter estimates and 95% credibility intervals (hereafter CI), we fit both models in WinBUGS using equivalent parameterizations of Eqns. (6) and (7). The number of years of protection affected the rate of change in coral cover. In the Caribbean, the rate of change in coral cover shifted from negative to positive at approximately 14 years (Fig. 4A). For the Indo-Pacific, we found that there were two distinct rates of coral cover change. The median of the posterior distribution of the changepoint model indicated that the transition between the two rates occurred at approximately 22 years. The location of the changepoint was likely the result of a major cohort of reefs, which had been protected for roughly 20 years, experiencing declines due to the 1998 El Niño event. Before the changepoint, the rate of change in coral cover shifted from negative to positive after 5 years and continued increasing until the changepoint at approximately 22 years (Fig. 4B). After the changepoint, the rate of change in coral cover became negative again, although not significantly different from zero (Fig. 4B).

***Bayesian fitting***

In order to obtain more realistic estimates of parameter precision, the final AIC best models were refit as Bayesian models in which uninformative priors were used for all parameters. Samples from the parameter posterior distributions were obtained using Markov chain Monte Carlo (MCMC) as implemented in WinBUGS 1.4.3 [6]. For the MPA-only model, a slightly informative prior was used for the breakpoint to bound the Markov chains away from zero. Three separate chains were run and mixing was assessed both graphically and analytically. Iterations were continued until all parameters yielded *R*^2^  values (the square root of the variance of the mixture of all chains divided by the average within chain variance) that were less than 1.1 for all parameters. The first half of each chain was discarded as a burn-in period and the remainder was thinned to minimize serial correlations.

***Model evaluation***

We also summarized the fit of our final model using a modification of *R*^2^, the coefficient of determination, generalized to multilevel models [13]. In a multilevel model, there are multiple sources of variation to be explained. At each level, we can write $\theta=\mu+\varepsilon$ where $\mu$ is the linear predictor, $\varepsilon$ is the random variation at that level, and $\theta$ corresponds to the individual data points (level 1) or a regression parameter (levels 2 and 3). Eqn. (4) is a generalized version of the equation above for multilevel models:

$R^{2}=1-\frac{\text{E}\left(\mathrm{Var}\left(\varepsilon_{i}\right)\right)}{\text{E}\left(\mathrm{Var}\left(\theta_{i}\right)\right)}$ (4)

In this notation, E is the expectation operator. The expectations in the ratio are calculated by taking the means of the posterior distributions of the variance obtained from a Bayesian fit of the model.

**References**

1. Bruno JF, Selig ER (2007) Regional decline of coral cover in the Indo-Pacific: timing, extent, and subregional comparisons. PLoS ONE 2: e711.

2. Mora C, Andrefouet S, Costello MJ, Kranenburg C, Rollo A, et al. (2006) Coral reefs and the global network of marine protected areas. Science 312: 1750-1751.

3. McMahon SM, Diez JM (2007) Scales of association: hierarchical linear models and the measurement of ecological systems. Ecology Letters 10: 437-452.

4. Pinheiro J, Bates D, DebRoy S, Sarkar D (2007) nlme: Linear and Nonlinear Mixed Effects Models.

5. R Development Core Team (2007) R: A language and environment for statistical computing. Vienna, Austria: R Foundation for Statistical Computing.

6. Lunn DJ, Thomas A, Best N, Spiegelhalter D (2000) WinBUGS - a Bayesian modelling framework: concepts, structure, and extensibility. Statistics and Computing 10: 325-337.

7. Lesaffre E, Rizopoulos D, Tsonaka R (2007) The logistic transform for bounded outcome scores. Biostatistics 8: 72-85.

8. Singer JD, Willet JB (2003) Applied longitudinal data analysis: modeling change and event occurrence. Oxford, UK: Oxford University Press.

9. O'Connor MI, Bruno JF, Gaines SD, Halpern BS, Lester SE, et al. (2007) Temperature control of larval dispersal and the implications for marine ecology, evolution, and conservation. Proceedings of the National Academy of Sciences of the United States of America 104: 1266-1271.

10. Akaike H (1973) Information theory and an extension of the maximum likelihood principle. In: Petrov BN, Csaki F, editors. Second International Symposium on Information Theory. Budapest: Akademiai Kaido. pp. 267-281.

11. Burnham KP, Anderson DR (2002) Model Selection and Multimodel Inference. New York: Springer-Verlag.

12. Toms JD, Lesperance ML (2003) Piecewise regression: a tool for identifying ecological thresholds. Ecology 84: 2034-2041.

13. Gelman A, Pardoe L (2006) Bayesian measures of explained variance and pooling in multilevel (hierarchical) models. Technometrics 48: 241-251.
