# Supplementary figures and images for: A Global Analysis of the Effectiveness of Marine Protected Areas in Preventing Coral Loss
Source: PLoS One. 2010 Feb 17;5(2):e9278. doi: 10.1371/journal.pone.0009278 (PMC2822846; doi:10.1371/journal.pone.0009278)

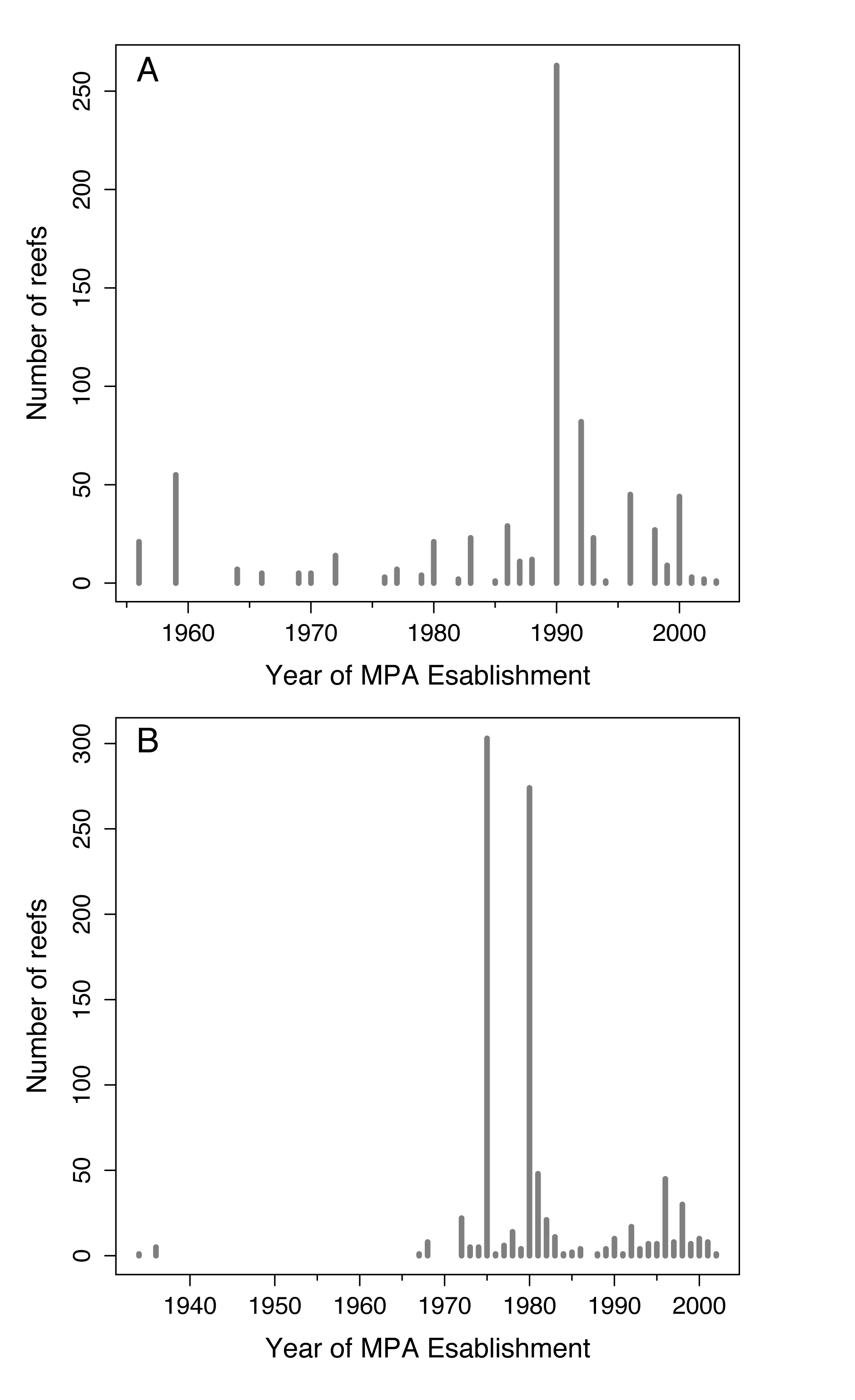

Supplement: Figure S1 — The number of reefs by the year of MPA establishment for the (A) Caribbean and (B) Indo-Pacific. (0.58 MB TIF) [file pone.0009278.s002.tif]

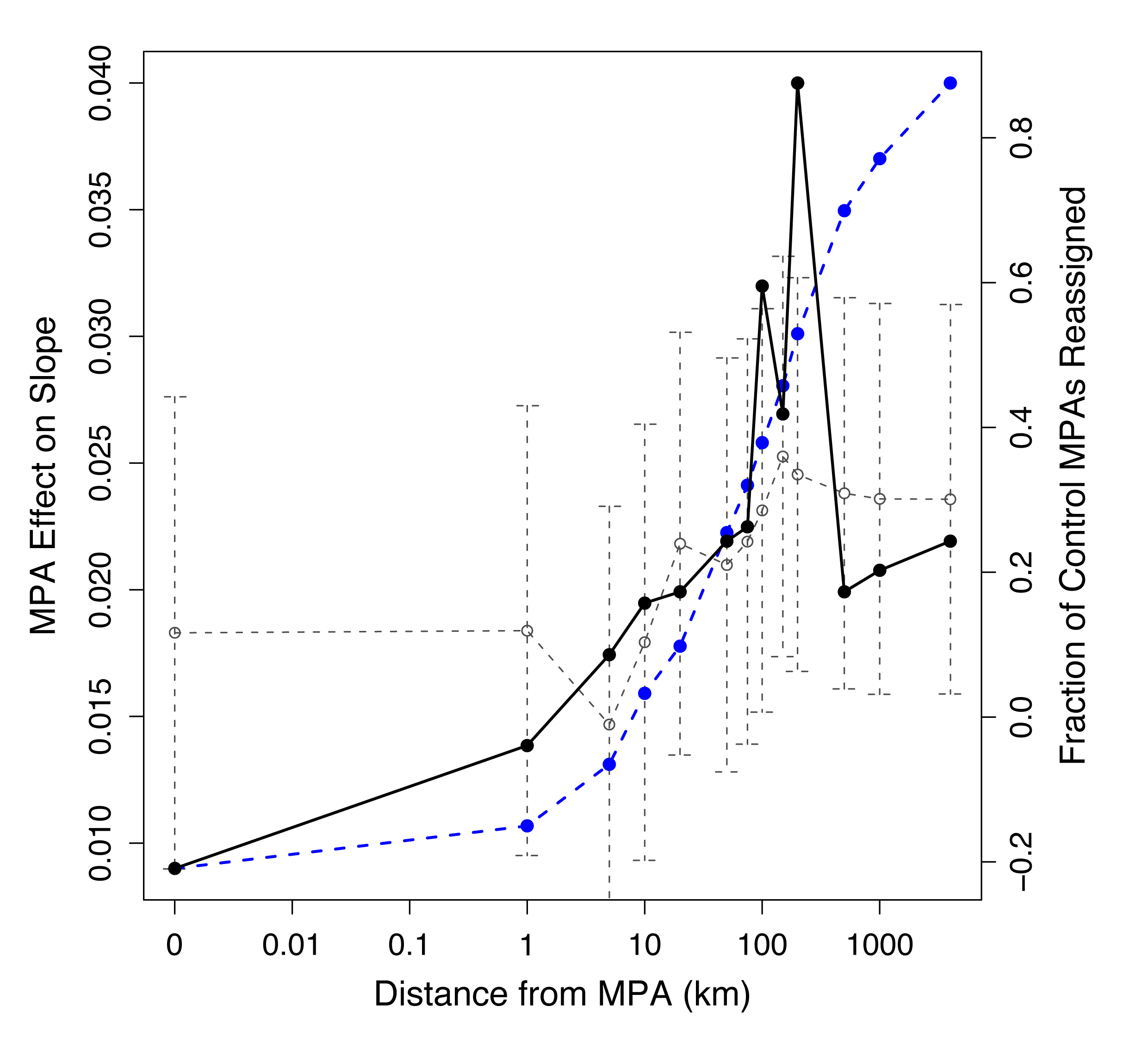

Supplement: Figure S2 — The relationship between the MPA effect on slope (change in coral cover) and the distance of non-MPAs surveys from MPAs. The loglikelihood (solid black line) is maximized at 200 km, where approximately 60% of the non-MPA data has been paired in a structural unit with MPA data (dashed green line). MPA effect on slope and confidence intervals (grey dashed line) do not vary significantly with distance. (0.54 MB TIF) [file pone.0009278.s003.tif]

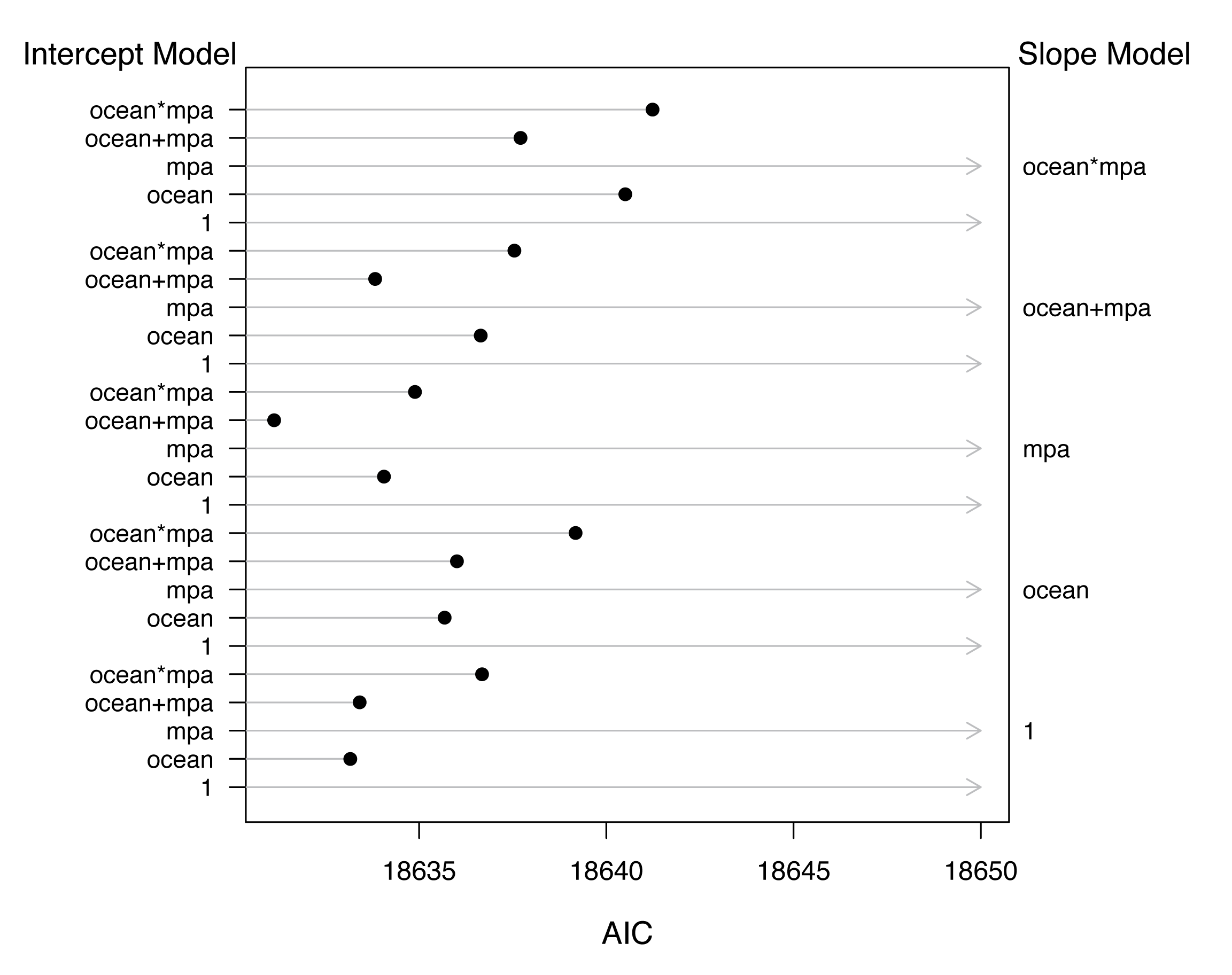

Supplement: Figure S3 — AIC values for all models examined. The best model is the one with the smallest AIC value. In this case, the best model is one in which MPA modifies the slope and intercept and ocean modifies the intercept only. Models with AICs that exceed 18650 are designated with arrows. (0.39 MB TIF) [file pone.0009278.s004.tif]

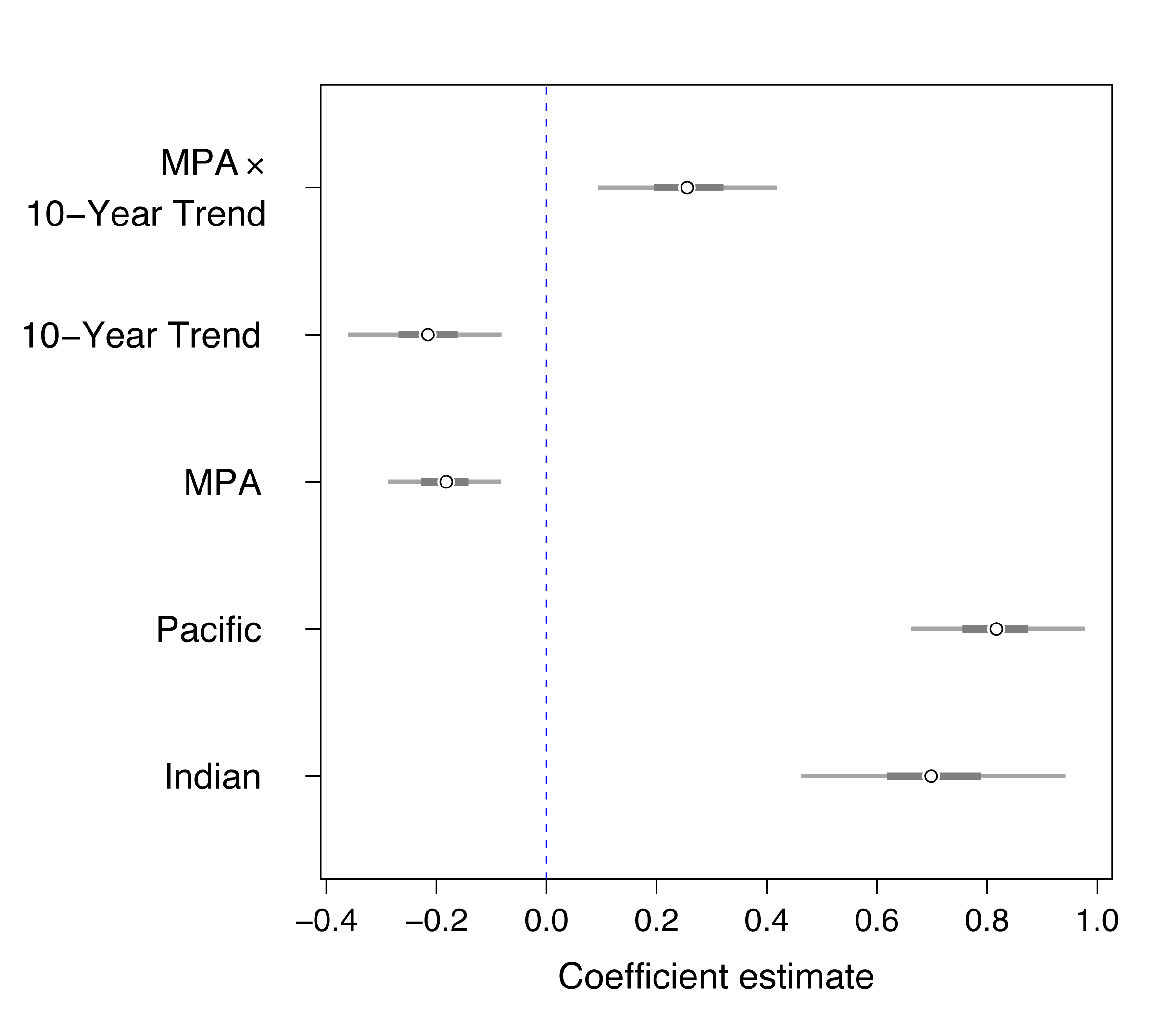

Supplement: Figure S4 — Coefficient estimates for the MPA versus non-MPA model. The 95% credibility intervals (thin light grey line) and the 50% credibility intervals (thick dark grey line) as well as point estimates (median) of the posterior distributions for all parameters in the MPA versus non-MPA model using a Bayesian approach to fit the model. There is a 95% probability that the true value lies within the 95% credibility interval. The MPA x 10-Year Trend term should be contrasted with the 10-Year Trend term, which is the trend for non-MPAs. The MPA x 10-Year Trend term is an effect and gets added to the 10-Year Trend term when MPA = 1 to obtain the trend for MPAs. (0.41 MB TIF) [file pone.0009278.s005.tif]

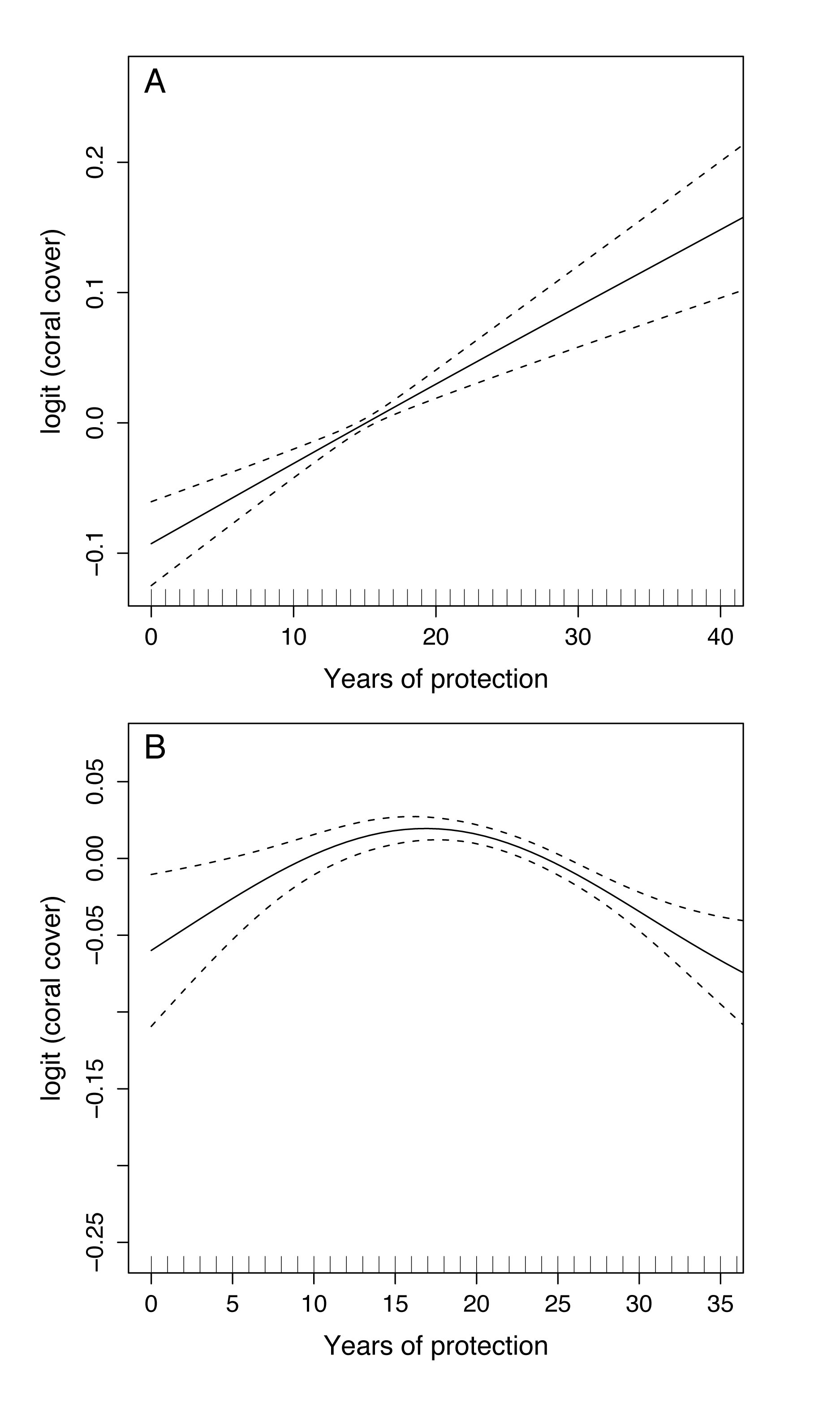

Supplement: Figure S5 — Generalized additive mixed models (non-parametric estimation) for the (A) Caribbean and (B) Indo-Pacific. There is no evidence of a changepoint in the Caribbean, but there is in the Indo-Pacific. The 95% confidence intervals are shown with dashed lines. The models have been smoothed with a 5-year running mean. (0.55 MB TIF) [file pone.0009278.s006.tif]
