# Supplementary material for: A Global Analysis of the Effectiveness of Marine Protected Areas in Preventing Coral Loss
Source: PLoS One. 2010 Feb 17;5(2):e9278. doi: 10.1371/journal.pone.0009278 (PMC2822846; doi:10.1371/journal.pone.0009278)
Supplement: Table S1 — R2 for MPA versus non-MPA model. NA denotes the lack of a predictor for the calculation of R2. (0.02 MB DOC) [file pone.0009278.s007.doc]

|  | $R^{2}$ **at each level** | | |
| --- | --- | --- | --- |
| **Model level** | $y$**(logit coral cover)** | **intercept** | **slope** |
| Level 1 | 0.84 | — | — |
| Level 2 | ­— | 0.46 | 0.45 |
| Level 3 | — | 0.39 | NA |
