# Supplementary material for: A Global Analysis of the Effectiveness of Marine Protected Areas in Preventing Coral Loss
Source: PLoS One. 2010 Feb 17;5(2):e9278. doi: 10.1371/journal.pone.0009278 (PMC2822846; doi:10.1371/journal.pone.0009278)
Supplement: Table S2 — R2 for MPA-only models in the Caribbean and Indo-Pacific. R2 can only be calculated at level 1 for these models. (0.02 MB DOC) [file pone.0009278.s008.doc]

|  | $y$**(logit coral cover)** |
| --- | --- |
| Caribbean | 0.91 |
| Indo-Pacific | 0.80 |
